# Generative modeling for RNA splicing predictions and design

**DOI:** 10.1101/2025.01.20.633986

**Published:** 2025-01-24

**Authors:** Di Wu, Natalie Maus, Anupama Jha, Kevin Yang, Benjamin D. Wales-McGrath, San Jewell, Anna Tangiyan, Peter Choi, Jacob R. Gardner, Yoseph Barash

**Affiliations:** 1 Department of Computer and Information Science, School of Engineering, University of Pennsylvania; 2 Department of Genome Sciences, University of Washington; 3 Department of Genetics, Perelman School of Medicine, University of Pennsylvania; 4 Department of Pathology & Laboratory Medicine, Perelman School of Medicine, University of Pennsylvania; 5 Division of Cancer Pathobiology, The Children’s Hospital of Philadelphia

## Abstract

Alternative splicing (AS) of pre-mRNA plays a crucial role in tissue-specific gene regulation, with disease implications due to splicing defects. Predicting and manipulating AS can therefore uncover new regulatory mechanisms and aid in therapeutics design. We introduce TrASPr+BOS, a generative AI model with Bayesian Optimization for predicting and designing RNA for tissue-specific splicing outcomes. TrASPr is a multi-transformer model that can handle different types of AS events and generalize to unseen cellular conditions. It then serves as an oracle, generating labeled data to train a Bayesian Optimization for Splicing (BOS) algorithm to design RNA for condition-specific splicing outcomes. We show TrASPr+BOS outperforms existing methods, enhancing tissue-specific AUPRC by up to 2.4 fold and capturing tissue-specific regulatory elements. We validate hundreds of predicted novel tissue-specific splicing variations and confirm new regulatory elements using dCas13. We envision TrASPr+BOS as a light yet accurate method researchers can probe or adopt for specific tasks.

## Introduction

**Figure 1.**
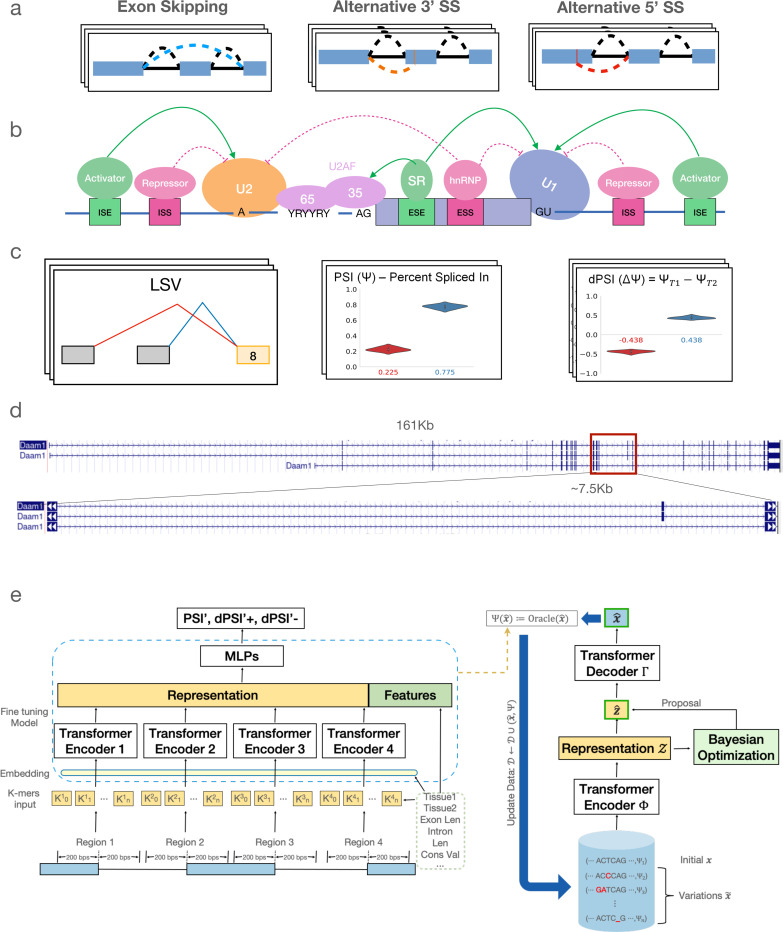
RNA alternative Splicing (AS) and its predictive generative modeling. **(a)** Basic types of AS. **(b)** Schematic of components involved in RNA splicing and its regulation. **(c)** Quantification of exon skipping events from RNA-Seq. PSI is used to represent their inclusion level, and dPSI is used to show the inclusion change across different conditions. **(d)** A genome browser view of an illustrative exon skipping event. The genomic regions spanned by cassette exons varies from tens to hundreds of thousands of bases. **(e)** The structure and flow of TrASPr and BOS. See main text for details.

Alternative splicing (AS) occurs when different mature mRNA transcripts are produced from the same gene by selectively including or excluding specific pre-mRNA exonic or intronic segments (see Figure 1a for illustrative examples). Over 90% of human genes undergo AS, with conservative estimates suggesting that at least 35% of human genes switch their dominant isoform across 16 adult tissues Pan et al. (2008); Wang et al. (2008); González-Porta et al. (2013). At the molecular level, AS can alter protein function, for instance, by removing a nuclear localization signal or modifying a binding domain within the encoded protein Smith and Valcárcel (2000); Licatalosi and Darnell (2010). Aberrant splicing has also been implicated in numerous diseases Singh and Cooper (2012). Consequently, a long-term objective for the RNA community has been to develop a predictive “splicing code” model capable of determining splicing outcomes based on genomic sequence and cell or tissue type Wang and Burge (2008). This long-term objective forms the focus of this work.

Decades of extensive research into the mechanisms of splicing regulation have identified hundreds of critical RNA-binding proteins (RBPs). These RBPs bind to exons and nearby intronic regions, typically within a few hundred bases of proximal splice sites, to modulate splicing in a condition-specific manner Fu and Ares Jr (2014a) (see Figure 1b for an illustration). However, translating this “parts list” of RBPs into a predictive splicing code Wang and Burge (2008) remains a significant challenge.

The advent of high-throughput RNA sequencing revolutionized the study of alternative splicing (AS), enabling researchers to detect and quantify thousands of AS events across diverse cellular conditions and tissues, as illustrated in Figure 1c. This advancement provided the data necessary to train predictive splicing codes using machine learning. Specifically, sequencing reads that span RNA segments joined by splicing (splice junction reads) are analyzed by dedicated algorithms such as MISO Katz et al. (2010), MAJIQ Vaquero-Garcia et al. (2016, 2023), and rMATS Shen et al. (2014) to quantify these AS events. The quantification of AS events, expressed as ‘percent spliced in’ (PSI), represents the ratio of isoforms (supported by junction-spanning reads) that include or exclude a specific RNA segment. Formally, PSI for a cassette exon e in cell type c can be denoted ${\text{Ψ}}_{e,c}\in [0,1]$, and changes in splicing for that exon between two cell types $c,c^{\prime }$, are denoted as dPSI ${\text{ΔΨ}}_{e,c,c^{\prime }}\in [-1,1]$. Depending on the task, these Ψ, ΔΨ values across numerous AS events and conditions can serve as labels to train machine learning algorithms.

As high-throughput splicing measurements became widely accessible, researchers were able to define a variety of prediction tasks based on this data. For instance, the first splicing code focused on qualitative changes in inclusion levels (“up,” “down,” or “no change”) for cassette exons across tissues, identifying associated regulatory mechanisms Barash et al. (2010). Subsequent studies shifted to related tasks, such as predicting the effects of genetic mutations on cassette exon inclusion Xiong et al. (2015); Cheng et al. (2021) or the creation and disruption of splice sites caused by mutations Jaganathan et al. (2019); Zeng and Li (2022). In this work, we address two tasks: quantitative tissue-specific splicing prediction for a given AS event and splicing sequence design. We demonstrate how these tasks are interconnected through deep generative models and illustrate their utility. We now proceed to describe each task and the related work in detail.

The tissue-specific prediction task involves predicting ${\text{Ψ}}_{e,c}$, ${\text{ΔΨ}}_{e,c,c^{\prime }}$ given the tissue or cell type and the genomic sequence of an AS event (e.g., cassette exon e). Early splicing code models relied on manually curated regulatory features from the literature to predict tissue-specific splicing changes Barash et al. (2010); Xiong et al. (2011, 2015). With advances in high-throughput splicing quantification, these earlier models—based on boosted decision trees and Bayesian neural networks—were replaced by deep neural networks, such as AutoEncoders, long short-term memory (LSTM) networks, and convolutional neural networks (CNNs) Jha et al. (2017); Bretschneider et al. (2018); Cheng et al. (2021); Zeng and Li (2022). Recent years saw a shift from using predefined features and relatively small models to large models that scan wide windows of genomic sequences, typically for predicting the effect of genetic variants on splicing outcomes. Such models do not assume a specific AS event type (e.g. cassette exon) but instead look for changes in splicing within a genomic window. Most notable is SpliceAI Jaganathan et al. (2019), which uses a CNN ResNet architecture, scanning 10 kilobases (kb) size windows across the genome to predict whether the center position is a splice site. More recently, Pangolin used the SpliceAI model architecture but trained it on several tissues and four species to predict tissue-specific PSI values $\left({\text{Ψ}}_{e,c}\right)$ Zeng and Li (2022). The emphasis on large models scanning genomic windows has extended to recent Large Language Model (LLM)-based approaches for various genomic tasks. Examples include DNABERT Ji et al. (2021), which pre-trains a BERT model on human DNA, SPLICEBERT Chen et al. (2023), which incorporates evolutionary conservation for splice-site prediction, and Enformer Avsec et al. (2021), which predicts gene expression across 200-kb windows. A notable recent addition is SpliceTransformer You et al. (2024), which we compare against and discuss in greater detail in the discussion section.

The tissue-specific splicing prediction task described above, as well as the application of LLM models to it, presents several challenges. One such challenge is the variability in the size of genomic regions containing cassette exons, which can range from a few hundred bases to several kilobases. As a result, applying ‘out-of-the-box’ transformer-based models like DNABERT Ji et al. (2021), which are limited to capturing sequences up to ∼500 bp in length, is unsuitable for cassette exons. Even larger models like SpliceAI and Pangolin, which are highly effective at splice site detection, span no more than 10 kb. In contrast, 24% of the cassette exons analyzed in this study span larger regions. Additionally, such regions may involve multiple exons and junctions, complicating the condition-specific prediction of PSI or dPSI. Another significant challenge is sample size. For any pair of cell types or tissues ($c,c^{\prime }$), the number of cassette exons exhibiting significant inclusion changes is typically limited to a few hundreds. Moreover, the RNA-binding proteins (RBPs) responsible for these changes are drawn from a pool of hundreds encoded in the human genome, and their specific contributions vary. Further complicating the task is the poorly defined nature of RBP binding sites, which have low information content and can either enhance or repress exon inclusion depending on their position Fu and Ares Jr (2014b) (see Figure 1b).

The second task we address in this work is splicing sequence design, a novel task introduced here. This task can be defined as generating a genomic sequence S with a specific splicing profile across various conditions {c}. For example, the goal may be to create a cassette exon e that is highly included in the Brain-Cerebellum but minimally included in other tissues. Importantly, the genomic sequence S may include intronic regions containing regulatory elements that influence the splicing of exon e. The sequence can also be based on an existing exon e that requires enhancement or correction through genetic editing. The allowed editing ‘budget’—such as the number of edits or total base changes—can be specified by the user to meet the requirements of a particular task. This flexibility is useful for applications like CRISPR editing or modifying exon inclusion using antisense oligonucleotides (ASOs) for therapeutic purposes. For instance, the model could be tasked with designing a new version of the cassette exon, restricted to no more than N edit locations and M total base changes.

Since the splicing sequence design task is novel, there are no prior implementations to reference. However, related work in molecular design, particularly in protein design, has seen significant progress using deep learning approaches (see Bepler and Berger (2021) for a review). For RNA design tasks, prior work has focused on areas such as optimizing untranslated regions (UTRs) in mRNA vaccines for enhanced expression Castillo-Hair and Seelig (2022); Leppek et al. (2022) and designing alternative polyadenylation sequences Bogard et al. (2019). The algorithms employed in these studies include genetic algorithms for 5’ UTR design Sample et al. (2019) and Deep Exploration Networks (DEN) Linder et al. (2019). However, DEN was developed for a distinct problem: 5’ splice site selection using a dataset of short synthetic sequences tested via MPRA. As such, these approaches differ substantially from the splicing sequence design task introduced in this work.

In the first part of this work, we address the challenges of tissue-specific splicing predictions by developing TrASPr, a multi-transformer-based model illustrated in Figure 1e (left). Compared to existing models, TrASPr offers several unique advantages and innovations.

First, instead of creating a large ‘foundation’ model that scans very wide genomic sequences and is ‘splicing agnostic,’ we develop a dedicated, lightweight model that leverages existing transcriptome annotations and current knowledge of splicing regulation. Decades of research indicate that splicing regulation is primarily localized around splice sites and influenced by condition-specific RBPs that mediate competition between splice sites. Based on this insight, we first pre-train a transformer to recognize splice sites. We then center a dedicated transformer around the splice sites of the cassette exon and its upstream and downstream (competing) exons (four splice sites total).

To account for features not captured by the local genomic sequence (e.g., intron and exon length, conservation), we incorporate additional genomic features. The representations learned by each transformer are combined using several joint multilayer perceptron (MLP) layers. This integrated model, TrASPr, is then trained on specific AS events (e.g., cassette exons) detected and quantified from the human transcriptome across different tissues.

By leveraging transcriptome annotations and quantifications, TrASPr can handle cassette exons spanning a wide range of window sizes—from 181 to 329,227 bases—thanks to its multi-transformer architecture. We first evaluate TrASPr using RNA splicing data from six human tissues (GTEx dataset), where it achieves state-of-the-art prediction accuracy compared to existing models. We also perform ablation studies to assess alternative architectures.

Next, we demonstrate TrASPr’s ability to detect regulatory elements, including tissue-specific ones, using diverse datasets. These include datasets involving four RBP knockdowns (KDs), a high-throughput mutagenesis assay introducing thousands of genetic variants, and a lower-throughput dataset for tissue-specific regulatory elements from a mini-gene reporter assay.

To illustrate the versatility of TrASPr, we showcase its application in two key tasks. First, we use it to predict novel tissue-specific splicing variations, which we confirm through targeted sequencing. Second, we validate TrASPr’s predictions of specific regulatory elements by targeting them with dCas13d.

TrASPr’s ability to predict tissue-specific splicing enables its use as a ‘teacher’ or ‘oracle’ for training a second deep generative model, which addresses the novel task of RNA splicing sequence design. To this end, we employ a variational autoencoder (VAE) transformer generative model, training it to structure its latent space representation using Bayesian Optimization (BO) techniques. Our Bayesian Optimization for Splicing (BOS) algorithm optimizes the VAE transformer under user defined sequence and splicing outcome constraints. We evaluate BOS-generated sequences against two baselines: random mutations and a previously proposed genetic algorithm. The results demonstrate that BOS more effectively mutates a given sequence, even with a limited number of mutations, to achieve a predefined tissue-specific splicing outcome. Additionally, we show that the mutations selected by BOS align well with experimentally validated mutations, providing further evidence of its utility. Finally, we conclude with a discussion of the potential applications of this approach, its limitations, and directions for future research.

## Results

### TrASPr offers improved prediction of cassette exon inclusion across diverse human tissues

**Figure 2.**
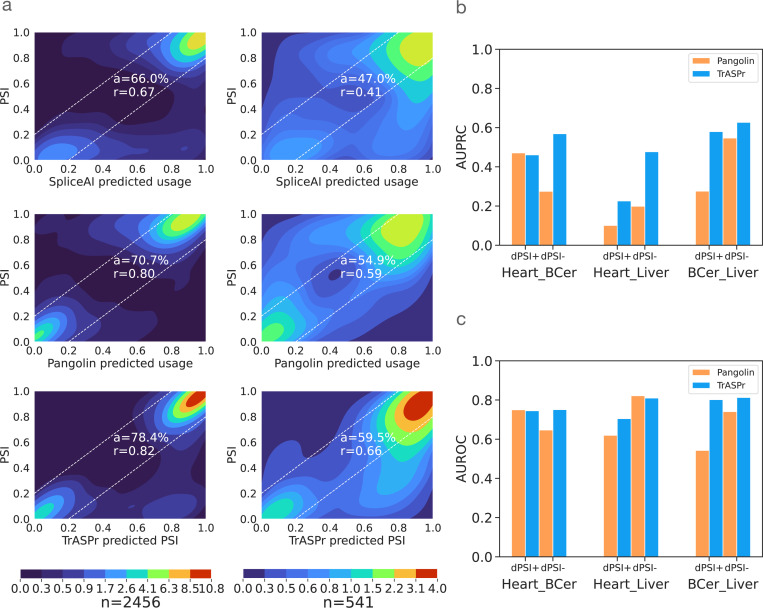
Comparison of PSI prediction results on GTEx dataset. **(a)** Heatmaps show the distribution of prediction vs. RNA-Seq values for all samples(left) and changing event samples(right) for SpliceAI (top), Pangolin (mid), and TrASPr (bottom). r is Pearson correlation, a is the proportion of predictions apprxomimately correct (within the dashed lines). **(b)** AUPRC for predicting events that are differentially included (dPSI+) or exlcuded (dPSI-) between two tissues. The tissue pair is denoted at the bottom, including Heart-Atrial Appendage, Brain-Cerebellum, and Liver. **(c)** Same as b above but for AUROC.

**Appendix Figure 1.**
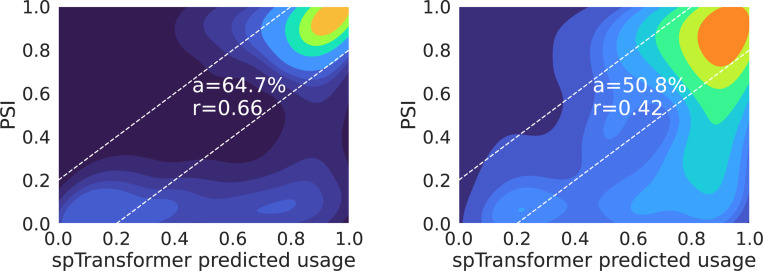
SpliceTransformer prediction results for GTEx dataset. Left is for all data samples and the right is for changing cases only.

To achieve accurate predictions for Ψ, ΔΨ across diverse human tissues, we start by pre-training a 6-layer 12-head BERT model on annotated 3’ and 5’ human splice sites. We then employ four such BERT models in the architecture of TrASPr shown in Figure 1e, training it on cassette exons quantified across six human tissues using GTEx V8 (see Methods for details). To assess TrASPr prediction accuracy, we compared it to the Pangolin Zeng and Li (2022) and SpliceAI Jaganathan et al. (2019) models. SpliceAI is considered the current state-of-the-art (SOTA) for predicting splice site strength, given a genomic window of 10 kb around it. While the model is agnostic to cassette exon definition or the cell type, it has been extensively used to approximate exon inclusion and predict mutations that disrupt splicing in patients. The more recent Pangolin model adopted the SpliceAI model for tissue-specific splicing, training it on data from four species, each with four tissues Zeng and Li (2022). For all three models, performance was evaluated on shared tissues and test chromosomes used by Pangolin Zeng and Li (2022) (see Appendix Section 2 for details). We assessed the performance of PSI prediction using two statistics: Pearson correlation (r) and the fraction of samples (denoted a) for which the model’s PSI prediction was close to the observed value (< 0.2 difference, *i.e.*, within the white dashed lines in Figure 2a). Treating SpliceAI as a baseline for SOTA non-tissue specific splice site strength prediction, we see in Figure 2a left column that it is able to achieve a=0.67, r=0.69. Its tissue-specific adaptation (Pangolin) achieves a=0.71, r=0.8, but TrASPr outperforms both with improved PSI approximation a=0.81, r=0.83. Still, those statistics are dominated by extreme values (close to 0 or 1) and the fact that most events do not change between tissues. Thus, when we repeat this analysis only for samples that are changing between two conditions (|ΔΨ|>0.15), performance degrades for all three models, but the differences between them become more striking (Figure 2a, right column). SpliceAI model is not tissue-specific and drops to a=0.47, r=0.41. As expected, the tissue-specific Pangolin model improves on SpliceAI to achieve a=0.55, r=0.59, but TrASPr significantly outperforms both with a=0.65, r=0.68. Finally, we also evaluated the recently published SpliceTransformer on this task, finding it performs similar to SpliceAI and worse than Pangolin (see Appendix Figure 1).

The results above calibrate current SOTA performance for tissue-specific PSI prediction and raise a related question: Even if the exact PSI prediction is not accurate, can these models accurately predict which cassette exons are being regulated in a tissue-specific manner? Answering this question has been the focus of the original splicing code models Barash et al. (2010); Xiong et al. (2011); Barash et al. (2013) and carries practical applications: Even if the magnitude of the change is not well calibrated, the models are likely able to identify regulatory features that are responsible for tissue-specific effects. In terms of assessing the models, this translates to measuring accuracy on the matching *classification* rather than regression task: For any two tissues $\left(c,c^{\prime }\right)$, correctly classify the exons that are significantly differentially included (${\text{ΔΨ}}_{e,c,c^{\prime }}\geq 0.15$, denoted dPSI+) and those that are significantly differentially excluded (${\text{ΔΨ}}_{e,c,c^{\prime }}\leq -0.15$), denoted dPSI-). Figure 2b,c summarizes the results on this task in terms of AUPRC and AUROC. We note that for this task, SpliceAI is irrelevant as its predictions are not tissue-specific. The improvements offered by TrASPr for this task are particularly striking in AUPRC, which is arguably more important given the extreme label imbalance, with an average improvement of 1.81 fold and a maximum of 2.4 fold over Pangolin.

### Assessing TrASPr components

**Appendix Figure 2.**
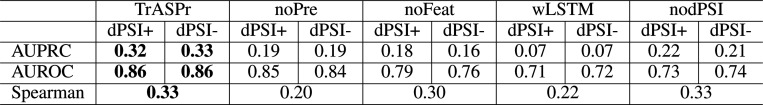
Ablation study. Left column (TrASPr) is the full model with pre-trained transformers. noPre - Same structure and input as TrASPr but trained from scratch. noFeat - same train/pretrain as TrASPr but without extra features. wLSTM - model with a bidirectional LSTM instead of Transformer and without the extra features. nodPSI - remove dPSIs in target function.

The improved performance by TrASPr naturally brings the question of which components of the model contributed to this improvement. We addressed this question through a series of ablation studies where we replaced the transformer with an LSTM (wLSTM), removed the additional features (noFeat), removed pre-training (noPre), and replaced the target function (nodPSI). The results, summarized in Appendix Figure 2, indicate each of those components significantly improved performance. Finally, we also tried to use existing DNABERT, a larger BERT model trained on the entire human genome, instead of our lighter transformer pre-trained on splice junctions. We found DNABERT to be highly unstable for this task, requiring further parameter exploration, and even then, performance degraded. These results suggest that careful pre-training can be beneficial even when a smaller model is used and that since the coding sequence is only a small fraction of the human’s DNA, the DNABERT may be learning dependency structures that are less relevant for the task at hand.

### TrASPr generalizes to unseen cellular conditions and other AS types

**Figure 3.**
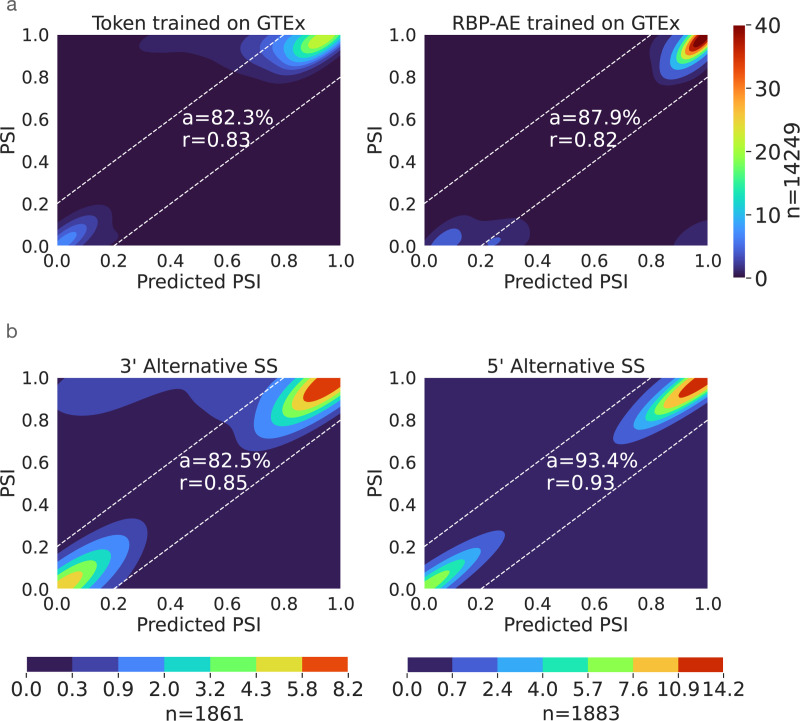
TrASPr prediction results in unseen conditions and alternative splice sites. **(a)** TrASPr was trained on GTEx 6 tissues and then tested on two cell lines in ENCODE (HepG2, K562). Left: The tissues were first represented as tokens, and new cell line results were predicted based on the average over conditions during training. Right: TrASPr used the RBP-AE learned representation to predict AS in the two ENCODE cell lines it never trained on. **(b)** Prediction accuracy of TrASPr when applied to alternative alternative 3’ (left) and alternative 5’ (right) splice sites.

**Appendix Figure 3.**
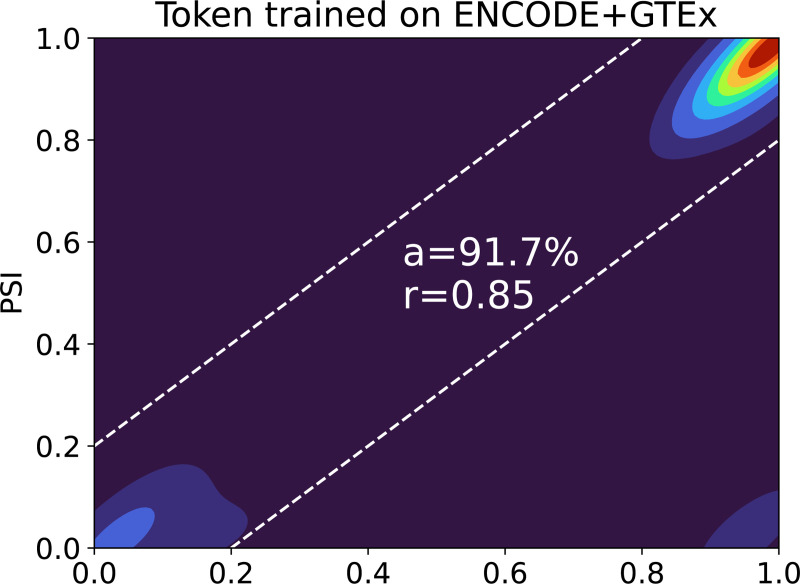
Prediction results for TrASPr with token tissue representation when both training and testing on ENCODE+GTEx dataset.

Existing condition-specific splicing prediction models only generalize over unseen genomic sequences but not cellular conditions. This limitation implies that predictions were only performed for conditions for which training data already existed, limiting the usability of the splicing codes. To address this, we introduced a new component into TrASPr involving an auto-encoder (RBP-AE) that learns a latent space representation for the tissue or cellular condition (see Methods). As shown in Figure 3a, this latent space representation allows TrASPr to generalize from the six GTEX tissues to unseen conditions (ENCODE cell lines), significantly improving prediction accuracy compared to TrASPr lacking RBP-AE (a=87.9% vs a=82.2%). This achieved accuracy is close to the one achieved when training on data from the ENCODE cell lines (a=91.7, See Appendix Figure 3).

Next, we wanted to assess the ability of our framework to generalize to other AS types. Notably, previous splicing codes focused solely on cassette exons. We therefore wondered whether the flexible architecture of TrASPr could be employed to predict other types of AS events. We used the same pre-trained BERT models and trained TrASPr on datasets for 3’ and 5’ splice site variations and observed similar performance (3’ r=0.85, 5’ r=0.93, see Figure 3b). In contrast, we observed significantly worse results for Pangolin and SpliceAI on this task(Pangolin: r=0.36, 0.31, SpliceAI: r=0.13, 0.10 for 5’ and 3’ splice sites dataset respectively). Taken together, these results indicate that TrASPr can generalize to both unseen cellular conditions and non-cassette exon AS events as well.

### Predicting the effect of regulatory elements and mutations

**Figure 4.**
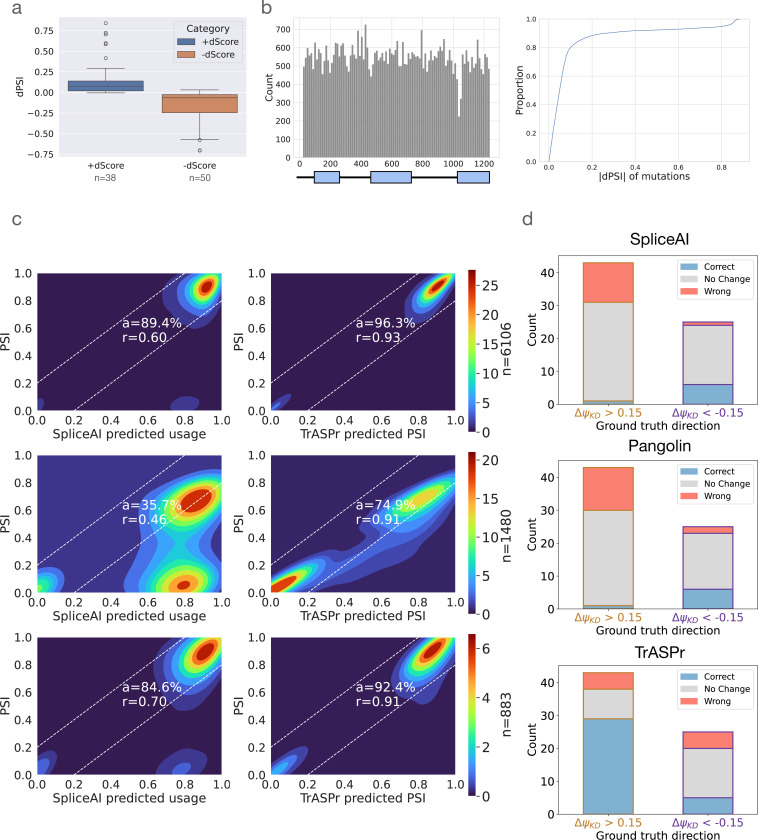
TrASPr prediction results on mutation effect. **(a)** Whisker plot for splice site mutation effect on predicted PSI when weak splice sites are made strong (blue, left) and when strong splice sites are made weak (brown, right). **(b)** Distribution of mutation positions in CD19 dataset (left) and the CDF of the marginal effect per each of those positions (right). **(c)** Heatmaps showing the performance of SpliceAI (left column) and TrASPr (right column) in predicting the effect of mutations shown in b, under two three settings: random 5-fold cross-validation (top row), random 5-fold cross-validation for changing mutations only (middle row), and single unseen mutation filter (bottom row). n indicates the number of cases in the test set. **(d)** Predicting the effect (dPSI direction) of RBPs KD by mutating their corresponding sequence motifs. Blue, grey, and red correspond to correct, no change, and opposite direction prediction, respectively.

Since TrASPr predicts PSI and dPSI directly from genomic sequences, it should be able to capture the effect of both tissue-agnostic and tissue-specific regulatory elements as well as the effect of genetic mutations on those. To assess TrASPr’s ability in such tasks, we first tested the effect of enhancing or degrading the core spliceosome 3’ and 5’ splice sites (see Appendix Section 2 for details). Figure 4a shows the results of this analysis for lowly included alternative exons whose splice sites were enhanced (left) and highly included exons whose splice sites were weakened (right). In both cases, the observed effect on PSI predictions is as expected, causing an increase (left) or decrease (right) in inclusion level. Notably, the magnitude of the change can vary greatly, and in general, weakening the splice sites tends to have a stronger effect. This result is to be expected as there are other elements (*e.g.*, branch point, Polypyrimidine tract) that can affect splicing, so weakening strong splice sites is likely to have a strong effect, but improving splice sites may not be sufficient to create strong exon inclusion.

**Appendix Figure 4.**
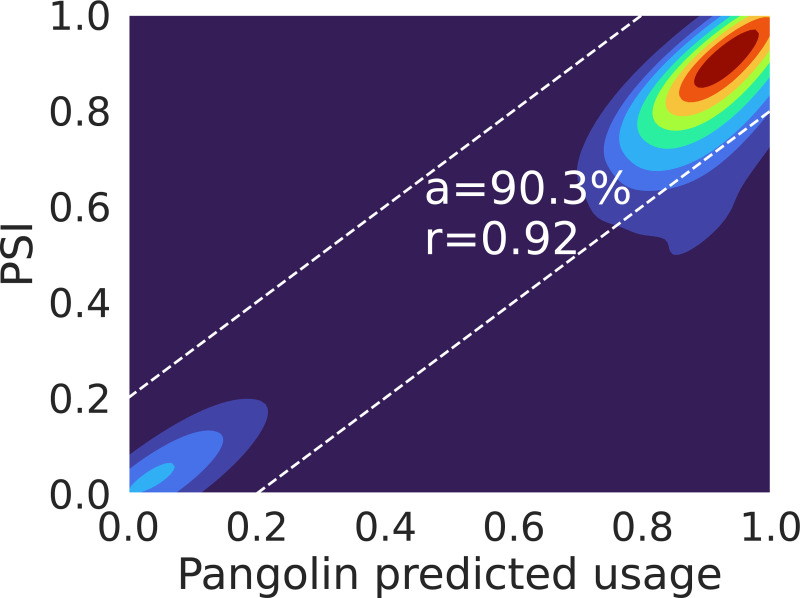
CD19 mutagenesis data results for Pangolin. Pangolin was re-trained and tested with the same data as TrASPr shown in the main text.

The above analysis serves as a qualitative sanity check that the model captures core splicing signals. However, a large-scale mutagenesis experiment can provide a more thorough quantitative analysis. For this, we used 6106 mutation combinations in exon 2 of CD19 and its flanking introns Cortés-López et al. (2022) and applied two testing schemes. The first is a standard 5-fold CV where 20% of combinations of point mutations were hidden in every fold while the second test involved ‘unseen mutation’ (UM) where we completely hide any sample that includes mutations in specific positions. We also noticed that while the mutations cover 1198 different genomic positions, most samples do not involve significant splicing changes (Figure 4b right). Thus, we assess performance on two sets of samples: all the data and only on the samples that cause significant changes. The results of this analysis are shown in Figure 4c. As expected, TrASPr performance degrades as the testing becomes more strict, yet it significantly outperforms SpliceAI in all settings. Pangolin performance is similar to SpliceAI (see Appendix Figure 4).

**Appendix Figure 5.**
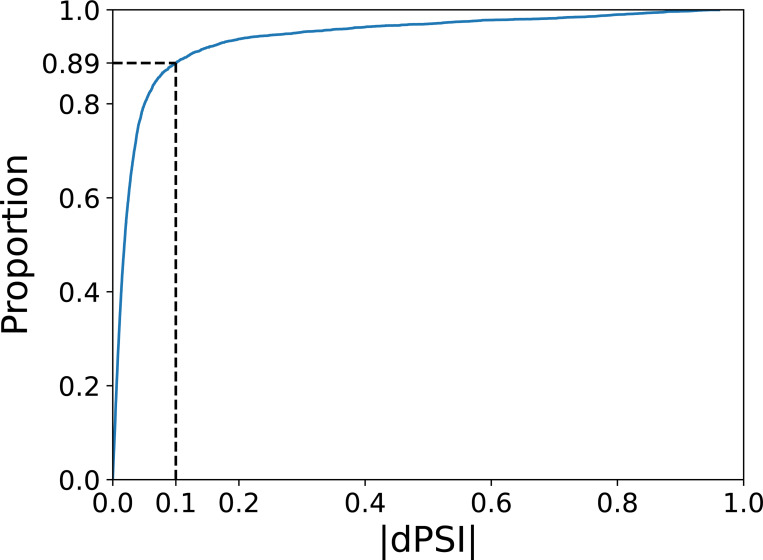
Prediction results of TrASPr compared to ground truth PSI on ENCODE dataset

A caveat of the previous evaluation is that it is not focused on RBPs and tissue-specific splicing regulatory elements, with strongly affecting mutations concentrated around splice sites. To assess TrASPr predictions for RBP regulatory elements, we turned to ENCODE data. First, we assessed whether TrASPr is able to predict exon inclusion in those new conditions accurately. As shown in Appendix Figure 5, TrASPr predicts Ψ within a 10% accuracy in almost 90% of the test cases, indicating excellent accuracy for the ENCODE cell lines. Next, we used RBP KD data Maurin et al. (2023); Van Nostrand et al. (2020) to compile a list of 68 cassette exons regulated by four well-studied, condition-specific RBPs (TIA1, PTBP1, QKI, RBFOX). For each of these targets, we recorded the experimentally measured effect (increased inclusion or exclusion) of the matching RBP Knockdown and compared it to the predicted effect by the model. Model prediction for the RBP KD effect was mimicked by mutating the genomic sequence corresponding to the RBP binding motif. Since most short sequence mutations are not expected to significantly alter PSI unless these ‘hit’ a core splicing signal, we set predictions to ‘no change’ when the dPSI effect was below the 95th percentile of effects observed by random mutations (see Data for details). Overall, TrASPr performed well on most positive effect cases but predicted around half of negative effects as no change. The correlation coefficient for the dPSI effects was 0.34 and the fraction of correctly called changes was over 50%, while only 20% were called incorrectly. In comparison, SpliceAI and Pangolin performed significantly worse (Figure 4d), with both predicting correctly only for 10.3% of the cases and predicting no effect for 70.6% and 67.6% respectively. These results indicate both SpliceAI and Pangolin struggle to capture condition-specific regulatory elements.

### TrASPr enables identification of new tissue specific splicing changes and regulatory elements

**Figure 5.**
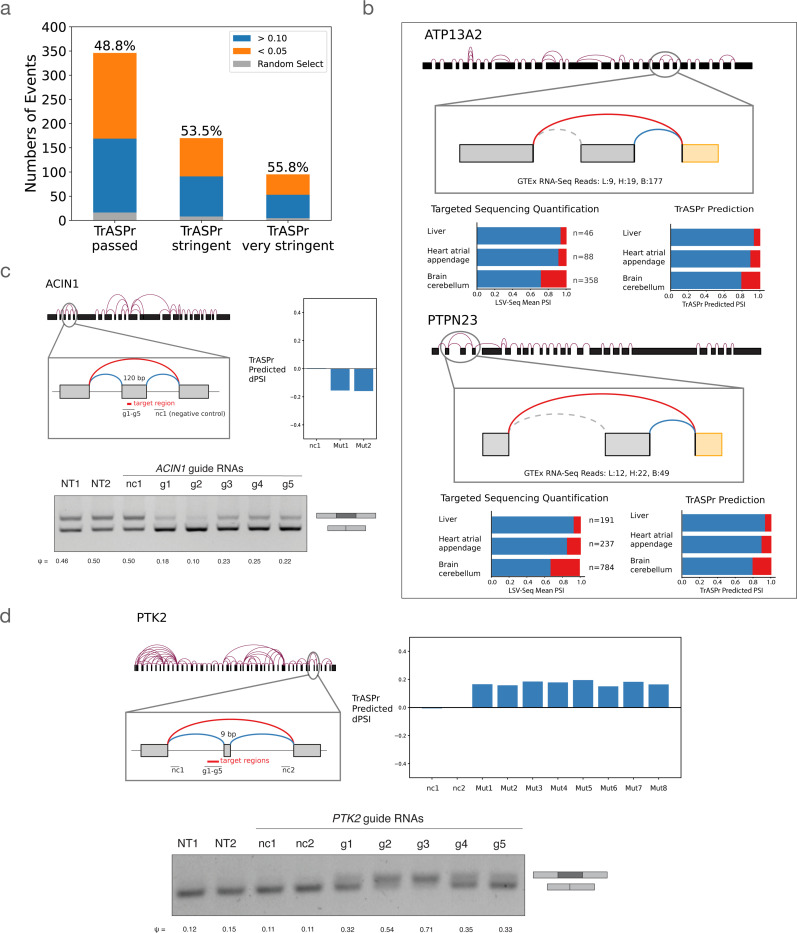
Experimental validations for TrASPr predictions. **(a)** Bar plot for the validation rate of low coverage AS events predicted by TrASPr to exhibit tissue-specific splicing between Brain-Cerebellum, Liver, and Heart-Atrial Appendage. Validation rate was between 48.8% to 55.8%, depending on the prediction stringency, discovering a total of 169 new tissue specific events. **(b)** Two examples of newly found tissue specific AS events from (a). For each case, the top graph illustrates the splicing context of the event. Two bar plots show the comparison between LSV-seq experimental results(bottom left) and TrASPr predictions(bottom right). **(c)(d)** Two AS events where specific regions were targeted by dCas13d including elements predicted by TrASPr to have significant regulatory effect and negative control regions. The bar plot(top right) shows the predicted inclusion level changes by TrASPr for 6b long windows in the tested region. Effects of dCas13d targeting were assessed by RT-PCR (bottom, NT = non-targeting, nc = negative control).

Given the strong performance we observed in predicting tissue specific splicing changes, we wanted to test whether TrASPr can be used to predict previously unknown splicing changes and regulatory elements. To do so, we employed LSV-Seq Yang et al. (2024), a recently-developed targeted sequencing method from our lab which allows for enrichment and quantification of splicing events. We hypothesized that we could use TrASPr to recover tissue-specific splicing changes from previously detectable but unquantifiable low-coverage splicing events identified from analysis of GTEx RNA-Seq experiments. We first created a list of such cassette events, then assessed those for tissue-specific splicing (ΔΨ>0.1) using TrASPr. Targeting the 787 predicted tissue-specific cassette exons predicted with LSV-Seq resulted in 558 events that had sufficient coverage (> 30 reads across at least 2/3 tissues) to be confidently quantified as changing (ΔΨ>0.1) or non changing (ΔΨ<0.05) compared to TrASPr predictions. Overall, TrASPr target-selection achieved good validation rates ranging from 48.8% to 55.8% validation rate depending on the threshold stringency, compared to an expected success rate of 4.7% for random exon selection (Figure 5a).

Overall, the above analysis led to the identification of 169 new tissue-specific cassette exons, two of which are highlighted in Figure 5b. One brain cerebellum-specific cassette exon skipping event predicted by TrASPr involved the ATP13A2 gene (aka PARK9). ATP13A2, a lysosomal transmembrane cation transporter, linked to an early-onset form of Parkinson’s Disease (PD) when loss-of-function mutations disrupt its function Dehay et al. (2012); Ramirez et al. (2006); Zhang et al. (2022). Here, we detect an exon in a cytosolic loop of the protein with elevated skipping in the Brain-Cerebellum. The major protein isoform with this exon skipped, which also contains variations in the C-terminal, is degraded by the proteasome after mis-localization to the endoplasmic reticulum (ER) membrane Ugolino et al. (2011). However, the specific function of this exon in the protein remains unknown. Interestingly, many PD-associated mutations degrade ATP13A2 through a similar mechanism Podhajska et al. (2012); Ramirez et al. (2006); Ugolino et al. (2011). But while ATP13A2 proteasomal degradation can have significant consequences for disease, it is unclear what role this process or its regulation by alternative splicing plays in normal tissue. A second example of validating Brain-Cerebellum-specific cassette exon skipping predicted by TrASPr involves the PTPN23 gene. PTPN23 is an essential gene with diverse molecular functions, including degradation of ubiquitinated proteins Doyotte et al. (2008); Ma et al. (2015), regulation of the SMN complex Husedzinovic et al. (2015), and regulation of neuron pruning Loncle et al. (2015). Proper regulation of PTPN23 expression is important and its dysregulation often causes disease in many tissues Gingras et al. (2009). PTPN23 haploinsufficiency or under-expression are associated with neurodevelopmental delays Bend et al. (2020), heart defects associated with cardiomyopathy Xu et al. (2024), and cancer growth Cao et al. (1998); Manteghi et al. (2016); Singh et al. (2023). Here, TrASPr predicts tissue specific splicing of an exon in PTPN23, whose skipping leads to a premature termination codon and nonsense mediated decay of PTPN23. This suggests a mechanism for tissue-specific regulation of PTPN23 expression, and could also be potentially used for therapeutic modulation of PTPN23 expression in disease.

Next, we turned to experimentally test specific regulatory elements. For this, we first trained TrASPr to predict differential splicing between two cell lines, HEK293T (embryonic kidney cell line) and SH-SY5Y (neuroblastoma cell line) and then selected specific positive (affecting splicing) and negative (non-affection) regions around the cassette exon for validation (see Methods). The results for validating such regions around two cassette exons are shown in Figure 5c,d where g1–5 are the sgRNAs used to target dCas13 region, and Mut1,2... are the mutations selected by TrASPr based on dPSI. For all sites tested, we observed a strong agreement with the computational predictions. Taken together, these results point to the usefulness of TrASPr in predicting tissue specific splicing and regulatory elements that have not been observed or detected before. This ability raises the question if we can utilize TrASPr to then design new sequences as well. We turn to tackle this question next.

### Assessing BOS sequence generation

In order to generate genomic sequence with a specific splicing profile, we use TrASPr to define a black-box objective function, and optimize it using Bayesian Optimization (BO). Specifically, using BO, we optimize over the space of possible RNA sequences to find sequences that successfully accomplish some task according to TrASPr (*e.g.*, increase exon inclusion) which serves as a teacher or oracle. BO is an iterative, model-based optimization procedure involving the use of a surrogate model (typically a Gaussian Process, or GP) to approximate the objective function and iteratively select the most promising candidate points to evaluate. In our case, we use a GP model to iteratively select candidate RNA sequences which are then evaluated using TrASPr. Note that GPs and other standard surrogate models cannot be defined directly over the structured, combinatorial space of all possible RNA sequences. We therefore use a special kind of BO called Latent Space Bayesian Optimization (LSBO). LSBO allows BO to be applied over structured search spaces by first learning a continuous, numerical, latent space representation of the structured space. To obtain a latent space representation of the RNA sequence space, we pre-train (unsupervised) a Transformer based variational auto encoder (VAE) model on a large set of RNA sequence data. We then define our GP surrogate model over the learned latent space and run LSBO. We refer to this method as Bayesian Optimization for Splicing, or BOS (see Methods for more details).

We assess the ability of BOS to generate RNA sequences for a variety of tasks and compare it to two baselines. The first baseline method randomly mutated 3, 6, 15 and 30-mers in different regions in the hope of achieving the desired effect. The second baseline is a genetic algorithm (GA) as in Sample et al. (2019), originally applied to design 5’ UTR sequences. In all cases, we assessed how many of the generated sequences matched the desired criteria (*e.g.*, increase exon inclusion) and what was the best scoring sequence found (*e.g.*, max dPSI achieved). Note that in these evaluations we assume the values predicted by TrASPr (PSI, dPSI) for a generated sequence are correct and only assess each algorithm’s ability to generate candidate sequences efficiently.

**Figure 6.**
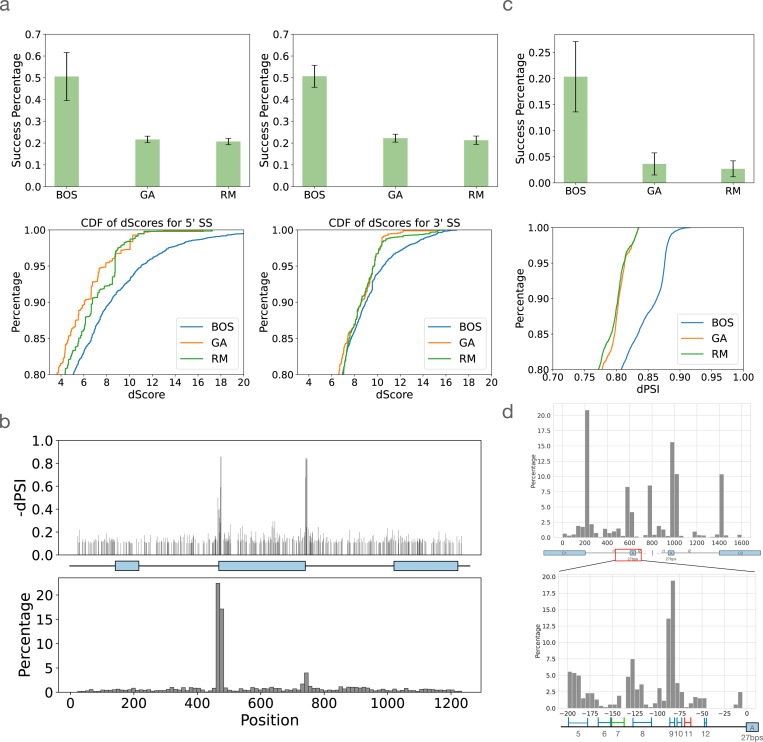
RNA design results by BOS **(a)** Results for the task of improving inclusion of weak cassette exons (n=8 exons). Top: Bar plots for success rate in achieving desired design task (increased inclusion). Error bars represent standard deviation over the set of exons tested. Bottom: CDFs over the best designed sequences (top 20%) by the MaxEnt splice site score change between the original sequence and proposed sequence. GA - Genetic Algorithm, RM - Random. **(b)** BOS generation results for CD19 mutation dataset. The positions mutated by BOS (bottom) capture regions close to the alternative exon splice sites whose mutations have strong marginal effects on inclusion levels (top). **(c)** Comparison of BOS, GA and RM on tissue-specific(Brain-Cerebellum) sequence generation. Different start sequences (n=10) are randomly chosen from cassette exons exhibiting low inclusion levels. Every algorithm is tasked with adopting the start sequence to achieve Brain-Cerebellum-specific high-inclusion (Ψ≥0.5 for Cerebellum, otherwise Ψ≤0.2) within 30 edits. Top: Success rate for this task. Bottom: The achieved improvement (dPSI) for the top 20% sequences generated by each algorithm. **(d)** BOS generation results for neuronal specific Daam1 exon 16. Bar plots indicate the distribution of hits where BOS mutated. The bottom plot is the zoom-in region of the top one. Regions that were validated experimentally by mutating them in a mini-gene systems are marked either blue (yes) or red (no) depending if TrASPr that teaches BOS is able to predict the effect of those segments. The green region indicates a region that doesn’t affect the inclusion level and is predicted correctly by TrASPr.

The first task we used to assess BOS was to improve inclusion levels (PSI) of weak cassette exons. Here, we started from cassette exons where either the 3’ or 5’ splice site was weakened and instructed BOS to improve inclusion levels. BOS was able to achieve a mean success rate of over 50% (Figure 6a) and mean increase of inclusion of 40%. In comparison both RM and GA achieved less than half of BOS success rate (∼ 21%) with lower inclusion levels. Indeed, when we used the MaxEnt Yeo and Burge (2003) algorithm to score the splice sites in the generated sequences we find BOS is able to produce sequences with significantly better splice sites (Figure 6a bottom).

While the above results indicated strong performance by BOS they relied on computationally assessing performance by TrASPr and the MaxEnt algorithm. To assess BOS generated sequences with respect to experimental evidence we employed the high-throughput mutagenesis CD19 exon 2 experiment Cortés-López et al. (2022). Overall, this experiment included mutations to 1198 positions spanning the entire cassette exon region as shown in Figure 4b. To assess site selection by BOS we computed the marginal effect observed per position when mutated and compared it against BOS frequency of selecting it. Plotting the location of mutated positions to decrease exon2 inclusion we see BOS ‘locks’ on the two areas proximal to the splice sites which have the largest marginal effect (Figure 6b). More generally, we find that BOS learns to favor more affecting positions in terms of their marginal dPSI effect, selecting the top 10% of affecting positions (marginal dPSI > 0.114) 31.2% compared to only 11.0% for positions at the bottom 10% (dPSI < 0.03, binomial p-value < 10^−4^).

Next, to assess ability to alter splicing events in a tissue specific manner, we supplied each algorithm with lowly included cassette exons and requested no more than 30 edits that will make those have a relative increased inclusion in Brain-Cerebellum compared to other tissues at least 30%. On this task, we take generated sequences with high inclusion level in Brain-Cerebellum (PSI> 0.5) and low inclusion in other tissues(PSI< 0.2) as successful. Notably, this is a much harder task compared to the non-tissue specific ones described before. Indeed, when assessing ten different lowly included exons as a starting point, TrASPr was only able to match the user constraints 20% of the times on average, but GA and RA exhibited a much lower success rate of approximately 4% (Figure 6c top). The differences in performance between the algorithms was even more striking when considering the best 20% of proposed sequences (Figure 6c bottom).

**Appendix Figure 6.**
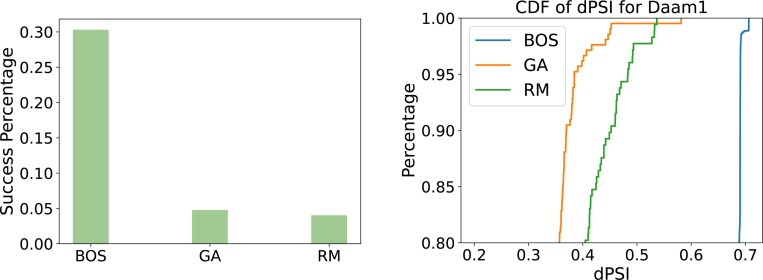
Generated sequences result for Daam1 gene exon 16, aimed to reduce inclusion in N2A cell line (dPSI>0.2) but not totally destroy the inclusion in other tissues(PSI>0.1)

Finally, to assess BOS generation with respect to experimentally tested tissue specific regulation we gave BOS the sequences proximal to exon 16 in the Daam1 gene and instructed it to reduce inclusion levels but without abolishing exon inclusion (PSI > 0.1). Exon 16 is a well studied, neuronal specific, micro-exon which has been shown to have high inclusion levels in Brain-Cerebellum and in N2A cell lines (Ψ = 0.66). As such, previous work using a mini-gene reporter assay in N2A cells mapped several regulatory elements in the upstream intron that affect its inclusion (Figure 6d, colored regions). On this task, the best random mutation setting (30-mers) successfully generated 177 out of 4392 sequences(4.03%) that increased inclusion by more than 0.2. The GA successfully generated 210 sequences (4.7%), while BOS generated 1331 successful sequences (30.3%) that matched the constraint of dPSI>0.2 (See Appendix Figure 6). Furthermore, BOS also significantly outperformed the two baselines in terms of the best candidate sequence generated (0.71 maximum dPSI, compared to 0.61 and 0.53 for the GA and the random sampling algorithms, respectively). Majority of positions BOS chose to edit were, as expected, around the splice sites (Figure 6d top). However, zooming in on the upstream intron (Figure 6d bottom), we found BOS repeatedly mutated the validated enhancing regulatory elements, avoiding the negative control region (green). As expected, BOS still failed to suggest editing the red region on which TrASPr itself fails, demonstrating that the generative process is inherently limited by the capabilities of the oracle.

Overall, our analysis of *in-silico* predictions and experimental assays indicate that BOS is able to efficiently capture regulatory elements in a given sequence, including both splice site signals as well as deep intronic elements, then capitalize on those to generate sequences matching a given splicing target function.

## Discussion

In this study, we offer two main contributions. First, we propose a new tissue-specific splicing code model, TrASPr. TrASPr’s architecture leverages the transformer attention mechanism while utilizing multiple transformers, each focused on specific genomic regions. This design ensures the model concentrates on areas most relevant to splicing regulation without requiring excessively large models.

We demonstrated that TrASPr significantly outperforms current state-of-the-art models in predicting PSI and dPSI across multiple datasets, even when those models used considerably larger genomic windows. Moreover, to our knowledge, this is the first demonstration of the ability to predict PSI and dPSI under previously unseen experimental conditions and to predict variations in both 3’ and 5’ splice sites. Using TrASPr, we generated predictions that were experimentally validated to identify tissue-specific splicing variations undetectable by previous RNA sequencing methods, as well as condition-specific regulatory elements.

**Appendix Figure 7.**
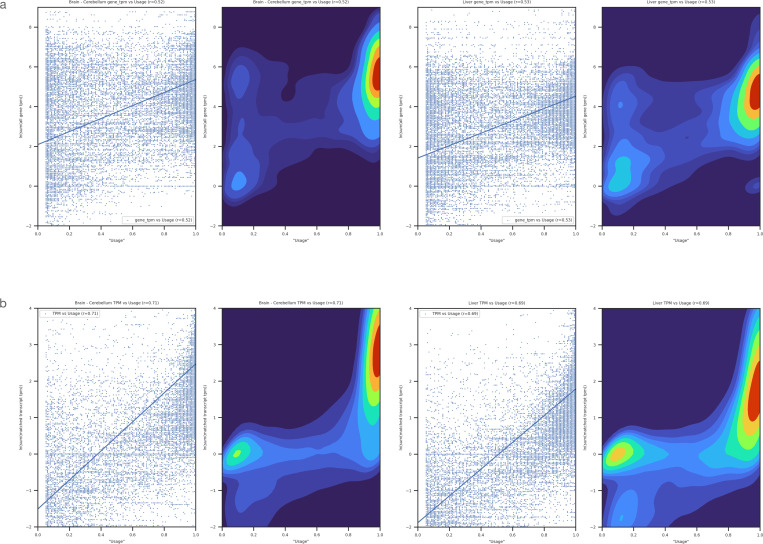
**(a)** Pearson correlation between gene expression (TPM) and splice site usage as defined by SpliceTranformer using GTEX Brain Cerebellum (r = 0.52, left) and liver (r = 0.53, right). **(b)** Correlation is further improved when considering the expression of only the isoforms that contain a specific splice site (cerebellum r = 0.71, Liver r = 0.69 right). TPM was computed using SALMON, only splice junctions from chromosome 8 were included to save on compute time.

**Appendix Figure 8.**
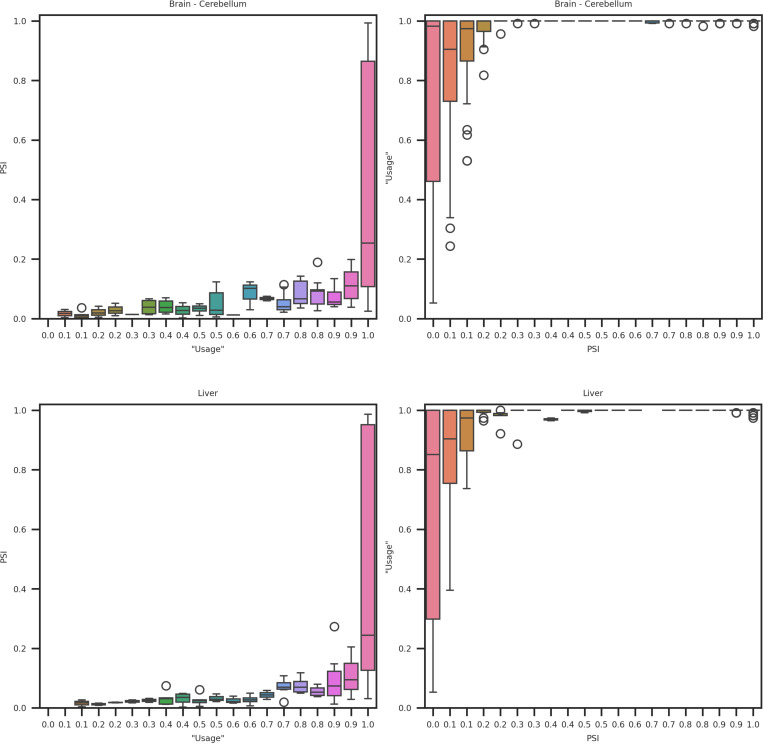
Tissue PSI values in GTEX samples (top - Cerebellum, bottom - liver) vs. SpliceTransformer usage. Correlation between usage and PSI is weak (Pearson r = 0.076 for Cerebellum, r = 0.074 for liver). Only shown are cassette exon PSI in chromosome 8 which were quantified by MAJIQ with high confidence and used as test data for all algorithms in the main text Fig2. When usage is high (left panels) PSI has the typical bimodal distribution such that it can either be very high or very low. Conversely, when PSI is low coverage can greatly vary between 0.05 (the threshold filter set by the SpliceTransformer authors) and 1 but when PSI is high the events are detected in almost all samples (usage close to 1).

**Appendix Figure 9.**
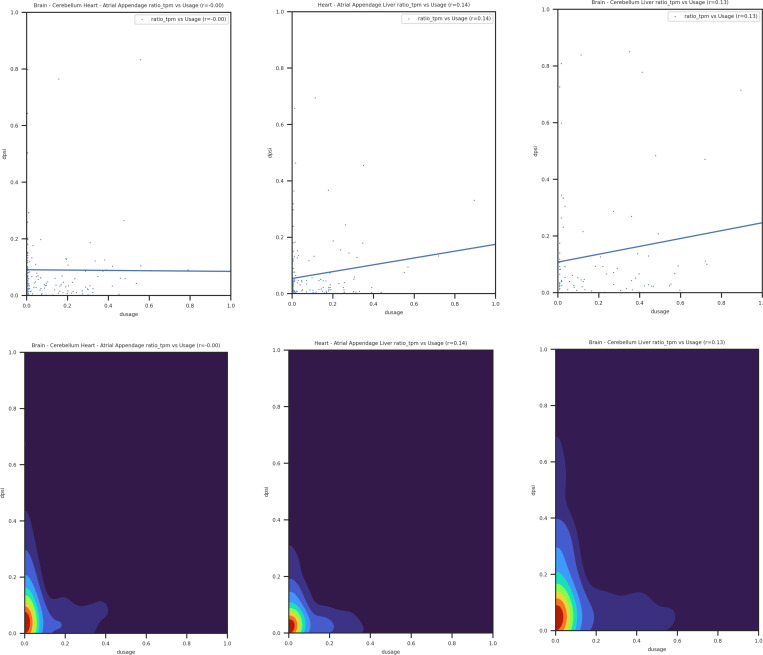
Differential usage (x-axis) vs. differential splicing (dPSI, y-axis) for cassette exons in chromosomes 7,8 assessed for the three GTEX tissue pairs used in the main text (Fig2): Heart_BCer, Heart_Liver, BCer_Liver. dPSI values are the same as those used to train and test all algorithms in the main text. Top: Scatter plot Bottom: matching heat map. Note that ∼ 80–90% of the samples with dPSI > 0.1 have dUsage ∼ 0 and therefore will not be captured by the SpliceTransformer target function which weighs samples by their dUsage. A few points exhibit high dUsage and high dPSI contributing to some correlation, with pearson correlation ranging from 0 to 0.14 depending on the tissue pair.

In terms of related work, the models closest to TrASPr are Pangolin Zeng and Li (2022) and the recent SpliceTransformer You et al. (2024), both designed to predict tissue-specific splicing. SpliceTransformer employs a similar approach to SpliceAI and Pangolin, scoring genomic positions as 3’ or 5’ splice sites within a window spanning 4,000 nucleotides upstream and downstream. Additionally, SpliceTransformer incorporates what the authors define as splice site “usage” across different GTEx tissues. Adding this usage statistic into its target function is designed to aid in tissue-specific splicing prediction. However, in practice we found that SpliceTransformer performed similarly to SpliceAI and worse than Pangolin (see Appendix Figure 1). A potential reason for this may lie in the SpliceTransformer’s “usage” based target function. “Usage” is defined as the fraction of GTEx tissue samples in which a splice site was identified. For instance, a value of 0.1 indicates the splice site was detected (i.e., supported by more than one read) in 10% of the tissue samples. Consequently, this addition to SpliceAI’s original target function correlates poorly with splicing or differential splicing quantification. Instead, “usage” primarily reflects splice site detection capability, which is largely influenced by read depth and gene expression levels (see Appendix Figure 7–9). We note that these results do not negate SpliceTransformer’s ability to detect mutations that disrupt or create splice sites for downstream analysis, similar to SpliceAI and Pangolin.

The second key contribution of this study is the formulation of RNA sequence design with specific splicing characteristics as a Bayesian optimization problem. We developed the BOS algorithm, which uses TrASPr as an oracle to tackle this design challenge by introducing biologically plausible mutations. Across various tasks, we showed that BOS effectively generates RNA sequences with the desired splicing changes, incorporating mutations that selectively create or disrupt core splicing signals and intronic regulatory elements as required.

There are several potential applications for the work proposed here. First, TrASPr provides a relatively ‘lightweight’ LLM that can be easily fine-tuned for additional cellular conditions of interest. This capability enables the detection of condition-specific splicing and associated regulatory elements in scenarios where experiments have not yet been conducted or in genes with low coverage. It can also be used to assess the effects of genetic variants, such as resolving undiagnosed cases of rare diseases. This application was recently demonstrated in Wagner et al. (2023), where SpliceAI emerged as a top-performing model. For RNA design tasks, BOS and similar algorithms offer valuable tools for synthetic biology studies and therapeutic applications. For example, these algorithms could guide the design of sequences to target with ASO (antisense oligonucleotide) therapies or prime editing approaches.

While the above applications are exciting, we acknowledge several limitations and areas for potential improvement in this work. First, although TrASPr demonstrated significant advancements in PSI and dPSI predictions, it remains far from perfect. Specifically, the current model was not optimized for predicting the effects of genetic variations and can only capture mutations within 200 bases of existing splice sites. Additionally, we have not evaluated TrASPr on complex splicing variations involving multiple alternative splice junctions. However, we note that such events are often simplified when analyzing changes between two cellular conditions Vaquero-Garcia et al. (2023).

As for the analyses conducted here, it is important to recognize that the labels used for evaluating prediction tasks are inherently noisy and limited in number. For instance, RNA-Seq quantification is prone to noise, as are RBP binding assays such as eCLIP. Furthermore, RBP regulatory motifs are relatively crude representations, meaning many targets may be missed. Changes observed upon RBP knockdown (KD) could also arise from indirect effects, such as another RBP influenced by the KD, or from other sequence motifs.

Finally, we note a recent trend in AI for genomics toward the development of large ‘foundation’ models that can be fine-tuned for specific tasks. At this time, it remains unclear in what tasks such models can deliver superior performance or when lighter, task-specific models may be more accurate or useful. A related question is how easily a model can be adapted to handle new tasks, conditions, or datasets Penzar et al. (2023).

In this work, we present a ‘middle ground’: leveraging the framework of LLM pre-training while constructing models tailored to specific tasks. This approach results in significantly lighter models that require fewer computational resources and are easier to adapt to new tasks. We envision TrASPr, paired with BOS for design tasks, as a ‘base’ model that can be readily fine-tuned for specific cell types, tissues, RBP knockdowns, and other conditions. Indeed, we demonstrated TrASPr’s ability to generalize to alternative 3’ and 5’ splice site usage and to unseen cellular conditions by utilizing the RBP-AE.

In summation, we are excited for the future of LLM for RNA prediction, optimization and design. We hope both the algorithms and the new RNA splicing design task will have a significant impact, serving the community on a variety of current tasks as well as a base for future developments.

## Methods

Our method, depicted in Figure 1e, involves two main components: A transformer-based splicing prediction model (TrASPr) and a Bayesian Optimization algorithm (BOS) to design RNA with desired properties. We now turn to describe the two modeling components in order.

### TrASPr

#### Pre-training RNA splice site BERT model

The foundation model for TrASPr is a 6-layer BERT model pre-trained on human RNA splice sites (Figure 1e). Following the pre-training step, as in Ji et al. (2021), TrASPr takes an RNA sequence converted to 6-mer tokens as input, but instead of using the BERT default maximum length, we feed the model with 400 bases long sequences where the splice site (either 5’ or 3’ splice site, as shown in the illustration) is in the center.

For pre-training, we follow BERT in randomly choosing 15% of tokens but additionally mask the surrounding six tokens for each one to account for our overlapping 6-mer tokenization. We used standard masked autoencoder training, calculating the loss from the original 15% of tokens that were masked. The model is pre-trained for 110k steps with a batch size of 40. The learning rate was set to 4e^−^4, and we used a linear scheduler with 10000 warm-up steps.

#### The TrASPr model and fine-tuning

The structure of TrASPr is depicted in Figure 1e. For any given AS event e, the input to TrASPr is a sequence composed of four sequences $S_{e}={\left\{S_{e}^{i}\right\}}_{i=1}^{4}$ such that each $S_{e}^{i}$ covers the exonic and intronic regions surrounding one of the four splice sites involved in the exon skipping AS event e. Each $S_{e}^{i}$ is fed through a matching pre-trained transformer $T^{i}$, which also accepts additional event features $F_{e}=\left\{F_{e,i}\right\}$ (see below). The latent space representation from each transformer $T^{i}$, captured by their respective CLS tokens, are concatenated together along with the feature set $F_{e}$ and fed into two hidden layer MLP with layer widths 3080 and 768.

##### Event features:

The additional feature set $F_{e}$ includes the exon and intron length information as binned tokens and the tissue type. We also include conservation values generated based on the PhastCons score Siepel et al. (2005) for each k-mer in the sequence. Exons generally have significantly higher conservation values, as these reflect selection pressure due to non-splicing related functions (coding for proteins). We, therefore, used the mean of all conservation scores to fill the exon regions but kept the original scores for the introns.

##### Supervision:

Since we are interested in learning splicing variations between different conditions we define target variables that force the model to learn those Jha et al. (2017). Specifically, based on the splicing outcome for an event e in two conditions c and $c^{\prime }$ the target variables include:

$$\;T_{{\text{Ψ}}_{e,c}}=E\left[{\text{Ψ}}_{e,c}\right]\;T_{\text{ΔΨ}{+}_{e,c,c^{\prime }}}=\left|\max\left(\epsilon ,E\left[{\text{ΔΨ}}_{e,c,c^{\prime }}\right]\right)\right|\;T_{\text{ΔΨ}{-}_{e,c,c^{\prime }}}=\left|\min\left(\epsilon ,E\left[{\text{ΔΨ}}_{e,c,c^{\prime }}\right]\right)\right|$$

Here $E\left[{\text{Ψ}}_{e,c}\right],E\left[{\text{ΔΨ}}_{e,c,c^{\prime }}\right]$ represent the posterior expected values for PSI and dPSI as estimated by MAJIQ from the RNA-Seq experiments Vaquero-Garcia et al. (2016). The $T_{\text{ΔΨ}{+}_{e,c,c^{\prime }}}$ target captures events with increased inclusion level between tissue c and ${\text{c}}^{\prime }$ while $T_{\text{ΔΨ}{-}_{e,c,c^{\prime }}}$ captures events with increased exclusion, incentivizing the model to focus its attention on splicing changes. To avoid the zero gradient issue, we use a random small number between 0.001 and 0.002 as ϵ. We use the cross-entropy loss function, which performed better than the mean-squared error loss during our ablation studies. In the fine-tuning step, we train the model with a 2e-5 learning rate and batch size of 32 for ten epochs.

#### Sequence design for splicing outcomes

Beyond supervised learning, we also demonstrate that TrASPr can be leveraged to solve sequence design problems. Given a sequence $S_{e}=\left(s_{1},\ldots ,s_{n}\right)$, TrASPr measures the probability that the splice site in the center of $S_{e}$ is included in some tissue c, ${\text{Ψ}}_{c}\left(S_{e}\right)$. This value can directly be used as the basis for optimization problems, where we seek to design new sequences ${\tilde{S}}_{e}$ that differ from $S_{e}$ only slightly but exhibit altered splicing outcomes. Formally, we define these optimization problems as:

(1) $$\;\underset{{\tilde{S}}_{e}}{\arg\min}{\text{Ψ}}_{c}\left({\tilde{S}}_{e}\right)\;\text{s.t.}\;\text{lev}\left({\tilde{S}}_{e},S_{e}\right)\leq \tau \;\;\text{or}\;\underset{S_{e}}{\arg\max}{\text{Ψ}}_{c}\left({\tilde{S}}_{e}\right)\;\text{s.t.}\;\text{lev}\left({\tilde{S}}_{e},S_{e}\right)\leq \tau$$

Here, $\text{lev}\left({\tilde{S}}_{e},S_{e}\right)$ denotes the Levenshtein distance between ${\tilde{S}}_{e}$ and $S_{e}$. Solving the minimization problem is equivalent to finding a small perturbation (up to edit distance τ) of $S_{e}$ that *reduces* inclusion in the target tissue c by as much as possible. The maximization problem corresponds to *increasing* inclusion. In practice, we add additional constraints that $\forall c^{\prime }\neq c$ and ${\text{Ψ}}_{c^{\prime }}\left({\tilde{S}}_{e}\right)$ cannot be reduced below 0.05. These additional constraints prevent an optimization routine from destroying splicing to such an extent that all inclusion levels are driven to zero.

To solve this optimization problem, we adapt recent work in latent space Bayesian optimization (LSBO) for black-box optimization problems over structured and discrete inputs Maus et al. (2022); Stanton et al. (2022); Gligorijević et al. (2021); Moss et al. (2020); Winter et al. (2019); SanchezLengelingandAspuru-Guzik (2018); Gómez-Bombarellietal. (2018); GriffithsandHernández-Lobato (2020); Grosnit et al. (2021). LSBO solves structured optimization problems using two primary components: (1) a deep variational autoencoder (VAE) model and (2) a Bayesian optimization routine.

##### Variational autoencoders for LSBO:

In LSBO, we train a VAE that assists in reducing the discrete optimization problem over sequences 𝓢 to a continuous optimization problem over the *latent space* of the VAE, $𝓩\subset {\mathbb{R}}^{d}$. Leveraging the same data used to train TrASPr, we train a 6 layer transformer encoder Φ:𝓢→𝓟(𝓩) and 6 layer transformer *decoder* Γ:𝓩→𝓟(𝓢) Vaswani et al. (2017). The encoder $\text{Φ}\left(S_{e}\right)$ maps sequences $S_{e}$ onto a distribution over real-valued, continuous latent vectors z. The decoder Γ(z) reverses this process probabilistically. The parameters Φ and Γ are trained so that we have $\text{Γ}\left(\text{Φ}\left(S_{e}\right)\right)\approx S_{e}$. Because we only care about the output sequence ${\tilde{S}}_{e}$, here we abuse notation and denote the most probable sequence output from the decoder as Γ(z). For optimization, the advantage the VAE provides is the ability to optimize over *latent vectors* z rather than directly over sequences $S_{e}$. This is because, for any z proposed by an optimization algorithm, we can evaluate ${\text{Ψ}}_{c}(\text{Γ}(z))$. We therefore search for a $\tilde{z}$ such that ${\tilde{S}}_{e}:=\text{Γ}(\tilde{z})$ is an optimal solution to the optimization problem.

##### Bayesian optimization:

With the optimization problem in Equation 1 reduced to a continuous problem over $\tilde{z}\in 𝓩$, we can now apply standard continuous black-box optimization algorithms. Bayesian optimization Garnett (2023) is among the most well-studied of these approaches in the machine learning literature. In iteration n of Bayesian optimization, we have a dataset ${𝓓}_{n}={\left\{\left(z_{i},y_{i}\right)\right\}}_{i=1}^{n}$ for which $y_{i}={\text{Ψ}}_{c}\left(\text{Γ}\left(z_{i}\right)\right)$ is the known objective value. We train a surrogate model of the objective function using this data–most commonly a Gaussian process Rasmussen (2003)–and use this surrogate to inform a policy–commonly called an *acquisition function*–that determines what latent vectors $z_{n+1}$ to consider next. This paper uses LOL-BO Maus et al. (2022) as our base off-the-shelf LS-BO algorithm. To accommodate the constraints in Equation 1, we modify LOL-BO to utilize SCBO Eriksson and Poloczek (2021) rather than TuRBO Eriksson et al. (2019) as the underlying optimization routine. As with the objective, the Levenshtein constraint is evaluated on the decoded latent vectors: ${\text{lev}}_{𝓩}\left(z,z^{\prime }\right)=\text{lev}\left(\text{Γ}(z),\text{Γ}\left(z^{\prime }\right)\right)$.

### Experimental validation

#### Re-Sequencing Low-Coverage Cassette Events with LSV-Seq

Subsequently, LSV-Seq was used to generate targeted libraries for this list of low-coverage cassette events prioritized for resequencing with TrASPr Yangetal. (2024). Briefly, primers for LSV-Seq were designed using the Optimal Prime algorithm, which uses machine-learning models to optimize primer sequences for both specificity and yield. To perform LSV-Seq, the resulting primers were synthesized as a single combined pool of over 1,000 primers and used in the first-strand reverse transcription reaction for RNA from each tissue of interest. Sequenceable libraries were created after additional reactions including second-strand synthesis, in vitro transcription, fragmentation, secondary reverse transcription, and final PCR amplification. The resulting libraries were aligned with STAR (cite). Quantification and visualization of psi values across tissues was performed with MAJIQ/VOILA (cite)

#### Targeting with dCas13d

In order to test regulatory elements predicted by TrASPr we first trained it to predict differential splicing between the two cell lines: SH-SY5Y and HEK-293T. Selecting cassette exons with high confidince predictions for cell-line specific splicing changes we then selected regions for experimental testing using the following procedure. We randomly mutated sequences in the alternative exon and flanking introns using a 6 bp sliding window. Each region in a window was mutated 5 times to avoid introducing new motifs and the average predicted PSI was compared to the wild type. Finally, we selected top target regions based on predicted dPSI.

For experimental validation, predicted regulatory or negative control sequences were targeted with dCas13d. HEK-293T cells were cultured in DMEM (Gibco, 10569010) supplemented with 10% (v/v) FBS (Thermo Scientific, A3160502) and 1x penicillin-streptomycin (Thermo Scientific, 15140122). For targeting, cells were co-transfected with vectors expressing dCas13d (pXR002, Addgene #109050) and guide RNA (cloned into a custom expression vector) using the CalPhos Mammalian Transfection Kit (Takara, 631312). Cells were collected two days later and RNA was extracted using the Direct-zol RNA Purification Kit (Zymo Research, D2052). RNA was converted to cDNA with the LunaScript RT SuperMix (NEB, M3010L) and PCR for the splicing event was performed with the Q5 Hot Start High-Fidelity 2X Master Mix (NEB, M0494S). PCR reactions were then visualized on a 2% agarose gel in 1X lithium boric acid buffer (Faster Better Media, LB10–1), stained with SYBR Safe (Invitrogen, S33102).

## Data

### Cassette exons quantification:

Appendix Section 1 details the process of detection and quantification of cassette exons that serve as training data for splicing code models. We measure splicing across c∈[1,…,C] conditions for events e∈[1,…,E]. Each AS event e has a sequence $S_{e}$ comprised of 4 different regions, each centered around the respective splice site $S_{e}=\left\{S_{e}^{1},S_{e}^{2},S_{e}^{3},S_{e}^{4},\right\}$. Similarly, each event has a set of associated features, such as exon length, conservation etc., denoted $F_{e}$. Splicing quantification for event e in condition c is denoted ${\text{Ψ}}_{e,c}\in [0,1]$ and differential splicing as ${\text{ΔΨ}}_{e,c,c^{\prime }}\in [-1,1]$ accordingly. However, we frequently drop the event e or condition c index for brevity.

### Pre-train and tissue-specific splicing data:

To pre-train the basic BERT RNA model, we first extract 1.5 million 400 bases long sequences around splice sites from the GENCODE human pre-mRNA transcripts database. For tissue specific splicing quantification we used the GTEx dataset Consortium (2020) from which we select six representative human tissues (Heart-Atrial Appendage, Brain-Cerebellum, Lung, Liver, Spleen, and EBV-transformed lymphocytes). In total, we collected 18278 cassette exons from the GTEx dataset with high-confidence quantification for their ${\text{Ψ}}_{e,c},{\text{ΔΨ}}_{e,c,c^{\prime }}$.

When training and testing on such data, care must be taken to avoid testing on similar cassette exons across genes in the same species (paralogs). We handle this issue by first hiding two chromosomes for testing (chr1,3,5,7,9 to have a fair comparison with Pangolin as in Zeng and Li (2022) and 8, 14 for the rest of experiments). Then, we discard test exons that are too similar to training exons (see Appendix Section 2 for details).

### Mutations and knockdown data:

To evaluate the capability of TrASPr and BOS to predict or suggest mutations, we curated four other datasets. The first dataset is the ENCODE database for RBP Knockdown (KD) in human cell lines Van Nostrand et al. (2020), where we focused on three well-studied RBPs (TIA1, PTBP1, QKI). This data resulted in a list of 59 putative RBP regulatory targets for which we could ‘remove’ the effect of these RBPs on the set of their AS targets by randomly mutating their identified binding motifs (see Appendix for more details). This set was then supplemented with nine validated targets of RBFOX from a recent study Maurin et al. (2023). In the prediction results, “change” and “no change” are determined based on a threshold set at the 95th percentile of observed effects from random mutations. To mitigate the positional effect of mutations, we utilized the same relative distance of the RBP binding sites from splice sites in our original set. For each position, we randomly select 100 events from the test set and performed five different random mutations. To ensure a fair comparison across different models, we evaluated all models on the same random mutation dataset and calculated their 95th percentile thresholds, which correspond to the following values. TrASPr: ΔPSI>0.019, SpliceAI: ΔUsage>0.043, Pangolin: ΔUsage>0.047. We note that these threshold values are specific to the exons and RBP sites we tested, aimed to create a uniform testing procedure for all methods. Finally, we included two additional datasets to assess splicing outcome predictions in the presence of genetic mutations. One is a recent high-throughput assay with 6106 mutation combinations around exon2 from the CD19 gene Cortés-López et al. (2022). This data is typical of assays that measure many mutations in a clinically relevant event, not necessarily tissue-specific ones. For capturing a tissue-specific event, we also included low-throughput experiments from a mini-gene reporter assay where the effect of mutating several regions upstream of the neuronal-specific exon 16 of the mouse Daam1 gene was tested Barash et al. (2010).

## Data Availability

Source code is available on GitHub. It contains sample data, source code of the model, and an executable notebook version of the paper to reproduce all paper figures. Trained models will be made available upon publication.

## Appendix

### Detection and quantification of alternative splicing from RNA Sequencing

This section is meant to help orient readers less familiar with RNA alternative splicing, its detection and quantification. Figure 1a shows common cases of alternative splicing. Here lines denote inronic regions of the pre-mRNA molecule that are removed while rectangles denote exonic regions that can be spliced together (dashed lines) to form the mature mRNA molecule. The most common type of AS event in human is cassette exons, as in Figure 1a left, where the middle exon can be either included or skipped. In many organisms, including human, exons are relatively short (median ∼ 145bp long in human) but introns can be much longer. For example, Figure 1d shows several mRNA isoforms of the Daam1 gene in mouse. Notably, the genomic window containing the isoforms is over 160Kb long, and the cassette exon region (red box) spans over 7.5Kb, illustrating the dimension of the problem. Detection and quantification of such AS events is mostly done nowadays with RNA sequencing, where the RNA is first fragmented, and then the fragments are sequenced using short reads (∼100b long) for one or two (paired) ends. These reads then need to be mapped back to the transcriptome or genome by dedicated mappers such as STAR Dobin et al. (2013). Only a minority of those reads are junction-spanning reads that span across segments spliced together. Algorithms such as MAJIQ used in this work then use those junction-spanning reads to quantify PSI or dPSI for specific segments or splice junctions as described in the main text. The results of this Ψ estimation is illustrated in Figure 1c as posterior distributions computed by MAJIQ for PSI or dPSI per junction in each LSV Vaquero-Garcia et al. (2016, 2023). A splicing code task is then to take in the genomic sequence in the region encompassing an AS event such as the cassette illustrated in Figure 1c and output a condition-specific PSI or dPSI for changes of exon inclusion between two conditions $c,c^{\prime }$. In practice, the outcome of splicing (PSI, dPSI) is the result of a complex multi-stage process illustrated in Figure 1b, where the spliceosome components recognize core signals (splice sites, branch point, poly-pyrimidine tract) while some of the hundreds of splicing regulatory factors, such as those of the hnRNP and SR families shown in Figure 1b, may bind in a condition-specific manner exonic and intronic areas around the splice sites involved, enhancing or repressing the inclusion of a specific segment in the mature mRNA (*cf* Fu and Ares Jr (2014b) for more detailed review).

For human tissue specific splicing we processed the GTEx dataset Consortium (2020) from which we select six representative tissues/conditions: Heart (Atrial Appendage), Brain (Cerebellum), Lung, Liver, Spleen, and EBV-transformed lymphocytes. These datasets were all processed uniformly using MAJIQ, as described in the Methods section, to detect and quantify cassette events.

Pangolin and SpliceAI models are unable to define specific splicing events such as cassette exons. Instead, they use a 10Kb sequence window and predict a ‘splice usage’ value for the 3’ or 5’ splice site candidate in the center position of the window. In the case of Pangolin, this is done using SpliSER Dent et al. (2021) to estimate a PSI value for each tissue. To make Pangolin and SpliceAI comparable for predicting cassette exon inclusion levels, we feed the 3’ and 5’ splice site of each alternative exon e. We tested different methods to calculate the results (e.g. using 3’ or 5’ splice site usage as PSI) and took the best method by calculating the average of these two. We also tried retraining Pangolin with PSI values from MAJIQ with similar results.

Finally, for the Transformer pre-training, we took the detected splice sites and created 400 bases long genomic region windows centered around these splice sites. These sequences were then converted into 6-mer tokens and fed as input to the BERT model.

### Cassette event filtering to avoid information leakage

The high number of cassette events in our data is partially due to the fact that the cassette exons extracted from MAJIQ’s splice graphs may overlap (*e.g.*, different splice sites used to define the skipped exon). This may be useful for training on diverse exon/intron definitions, but care must be taken to avoid information leakage to the test data. This is especially important for large models that can easily memorize genomic sequences Schreiberetal. (2020). In addition to overlapping cassette exon events, the human genome contains many similar genes, or paralogs, that were derived from a common ancestral gene. Both exons and splicing patterns may be similar between paralogs. To avoid these potential information leakage hazards, we first hide chromosomes (chr1,3,5,7,9 for experiments involved with Pangolin as in Zeng and Li (2022) and 8, 14 for the rest of experiments) for testing, then discarding test events similar to our training set. For this, we follow the procedure used in Jha et al. (2017), which controls for overlapping events and filters for events where the exons are similar. As in Jha et al. (2017) the sequence similarity was assessed using BLAT Kent (2002) with filters to identify the difference in length and the estimated similarity p-value. (BLAT settings maxLenDiff=5, minPval=0.0001 and minIdentity=95). This accounts also for short exons with high similarity but enough divergence relative to their short length not to achieve a significant p-value.

### Splice site swap and disruption for RNA sequence design

The splice site plays a critical role in alternative splicing. Identifying and then restoring them represents a crucial milestone for TrASPr and BOS. To assess how TrASPr responds to modifications in splice site strength, we scored all donor and acceptor splice sites using MaxEnt Yeo and Burge (2003). Based on these scores, we classified splice sites as strong or weak by selecting the top 15% (5’ score > 10.17, 3’ score > 10.83) and bottom 15% (5’ score < 5.95, 3’ score < 5.52) from the distribution of splice site scores in the test set. For low-inclusivity sequences (PSI < 0.15), we substituted their 3’ and 5’ splice sites in the middle exon with strong splice sites. Similarly, for high-inclusivity sequences (PSI > 0.85), we replaced the existing splice sites with weaker ones. Our goal was to observe TrASPr’s predicted changes in PSI before and after each replacement.

After confirming TrASPr’s ability on weak and strong splice site recognition, we further designed the experiment to test if BOS is able to recover the disrupted splice sites. In this case, we followed the same strategy to select high-inclusive sequences and strong splice sites. These strong 3’ or 5’ splice sites were disrupted through random mutations. Then, we randomly selected eight sequences each for the 3’ and 5’ splice sites as starters for generative models aimed at increasing PSI. In the end, the successfully generated sequences will be scored by MaxEnt to determine if their PSI is increased by generating strong splice sites.

### Pangolin and SpliceAI predictions on alternative splice sites

To evaluate if these methods are able to detect the tissue-specific alternative splice sites, we generated two datasets for 3’ and 5’ alternative splice sites, respectively. For each alternative splice site, we first scan for the ones from the MAJIQ splice graph. If no qualified alternative splice site for the chosen tissues exists, other real alternative ones that are expressed in other tissues will be selected based on Ensembl annotation and MAJIQlopedia. These non-expressed alternative splice sites are assigned with random small reads to calculate their PSIs. In the end, we got 1883 events for 5’ and 1861 events for 3’ splice site dataset.

Again, we applied the same data-splitting strategy for SpliceAI and Pangolin and tested different approaches to calculate the prediction results. However, both methods yielded significantly worse results for these two datasets(SpliceAI pearson≈0.15, Pangolin pearson≈0.22 for both datasets). Even when evaluating only the real subset for chosen tissues, their best performance remained substantially lower than TrASPr(best pearson≈0.28). This suggests that these methods for predicting splice site usage struggle to accurately identify tissue-specific alternative 3’ and 5’ splice sites and assess their relative inclusion.
